# Impact of Several Green Manure Species on the Physicochemical Characteristics, Enzymatic Activities, and Microbial Community Composition of Soils Under Protected Cultivation

**DOI:** 10.3390/plants15131965

**Published:** 2026-06-25

**Authors:** Jiahui Yu, Ke Xu, Zhengpeng Li, Xiaojun Wang, Qingbiao Yan, Kaibin Qi, Tianlong Chen, Mei Han

**Affiliations:** 1 College of Agriculture and Animal Husbandry, Qinghai University, Xining 810016, China; yujiah16@163.com (J.Y.); xk9417@126.com (K.X.); 2 Academy of Agriculture and Forestry Sciences, Qinghai University, Xining 810016, China; lipengzheng131@163.com (Z.L.);

**Keywords:** green manure, soil enzyme activity, microbial community, continuous cropping, soil amelioration

## Abstract

To evaluate the ameliorative effects of different green manure crops on continuously cropped protected pepper soil and to identify suitable green manure species for plateau-protected cultivation systems, a one-factor randomized complete block design was conducted with five treatments: common vetch (L1), pea (L2), hairy vetch (L3), radish (L4), and a control without green manure (CK). Soil physicochemical properties, enzyme activities, and microbial community composition were determined at the full-bloom stage before green manure incorporation. Compared with CK, L1 reduced soil pH from 8.63 to 8.34 and decreased total salt content by 45.5%, increased alkali-hydrolyzable nitrogen by 40.93%, and significantly enhanced catalase activity. L3 increased available phosphorus by 23.72% and urease and sucrase activities by 71.32% and 56.31%, respectively, while significantly affecting fungal β-diversity and community composition. Community composition analysis showed that L3 increased the relative abundances of the bacterial genus *Rhizobium* and the fungal genus *Rhizophagus*, while reducing the relative abundance of *Ascomycota* and several potentially pathogen-associated fungal taxa. Redundancy analysis and Mantel tests indicated that bacterial community composition was mainly associated with soil total salt content, alkaline phosphatase, and available phosphorus, whereas fungal community composition was more closely related to urease and alkaline phosphatase. Random forest analysis and partial least squares path modeling further suggested that sucrase, urease, and catalase were important factors closely associated with changes in the soil quality index (SQI). Overall, common vetch performed better in reducing soil salinity, increasing alkali-hydrolyzable nitrogen, and improving the soil quality index and may therefore be considered a suitable green manure species for improving continuously cropped protected pepper soil on the Qinghai Plateau. Hairy vetch showed advantages in increasing available phosphorus and regulating fungal community composition, indicating its potential suitability for protected soils with limited phosphorus availability.

## 1. Introduction

Protected vegetable cultivation, characterized by year-round production and high output efficiency, has become an important development model for plateau-featured agriculture [1]. However, with the extension of cultivation years and increasing cropping intensity, soil degradation under protected cultivation has become increasingly prominent. This degradation is mainly manifested as the deterioration of soil physicochemical properties, salt accumulation, nutrient imbalance, and the decline of soil ecological functions under continuous cropping conditions [2]. Pepper is one of the major economic vegetables cultivated in Qinghai Province. However, its industrial development has long been constrained by continuous cropping obstacles due to limited arable land resources, highly intensive protected cultivation, and restricted cropping arrangements [3]. Long-term monoculture can lead to soil structural degradation, the accumulation of salts and autotoxic substances, nutrient imbalance, and disruption of the soil microecosystem, thereby increasing the risk of enrichment of potentially harmful microorganisms and soil-borne disease occurrence, while reducing soil ecological functioning and the sustainability of crop production [4]. Soil microbial communities are essential components of soil ecosystems, and their composition and structure can reflect soil fertility status and are involved in nutrient transformation, organic matter decomposition, carbon, nitrogen, and phosphorus cycling, plant growth regulation, and soil-borne disease suppression [5]. Previous studies have suggested that an imbalance in soil microbial community structure is one of the key ecological mechanisms underlying continuous cropping obstacles [6]. Therefore, regulating soil microbial community structure and promoting nutrient cycling and organic matter transformation have become important research directions for alleviating continuous cropping obstacles under protected cultivation and improving soil quality. In recent years, studies on sustainable agricultural management have also emphasized that the stability of crop production systems should be improved through coordinated strategies, including efficient nutrient utilization, alleviation of plant nutrient stress, and maintenance of soil ecological functions [7].

Green manure is an internal biological resource with relatively low external inputs. In addition to providing organic residues and nutrient substrates, green manure cultivation can reduce nutrient losses by decreasing erosion and nutrient leaching, thereby contributing to more sustainable nutrient cycling in agricultural systems [8]. Previous studies have shown that green manure cultivation and incorporation can reduce soil salinity and pH while increasing soil organic matter, total nitrogen, and total phosphorus contents [9,10]. The cultivation of green manure during the winter fallow period can also increase soil organic matter levels and improve soil aggregate structure [11]. In addition, root exudates and decomposition products derived from green manure can provide carbon sources and nutrient substrates for soil microorganisms, thereby regulating the soil microecological environment [12,13]. For example, green manure treatments may increase the relative abundance of microbial taxa associated with organic matter decomposition, promote the transformation of plant-derived materials into available nutrients, and help maintain the stability of microbial communities involved in nitrogen cycling [14].

In protected continuous cropping systems, long-term green manure incorporation may also alleviate the decline in soil ecological functions caused by continuous cropping by altering the composition of rhizosphere soil metabolites, enriching potentially beneficial microorganisms, and improving soil chemical properties [15,16,17]. These studies indicate that green manure has considerable potential for soil improvement and microecological regulation. However, most existing studies have focused on low-altitude open-field systems or conventional crop rotation systems, whereas systematic research on how green manure regulates soil physicochemical properties, enzyme activities, and microbial community structure in continuously cropped protected vegetable soils on the plateau remains limited. Qinghai is characterized by a cold plateau climate, large diurnal temperature variation, alkaline soils, strong evaporation, and freeze–thaw alternation. Protected cultivation further creates a relatively enclosed environment with high humidity, intensive continuous cropping, and concentrated nutrient inputs. This unique soil ecosystem, shaped by the combined effects of plateau environmental conditions and protected cultivation, makes it difficult to directly extrapolate findings from low-altitude plain regions. Therefore, an independent investigation of continuously cropped protected pepper soils on the Qinghai Plateau is necessary.

Different green manure crops differ in root morphology, nutrient release characteristics, and rhizosphere exudation patterns, and these differences may lead to distinct effects on soil quality and microbial communities [18]. Previous studies have mostly focused on a single green manure crop or combinations of two species, while comparisons among different functional types of green manure within the same plateau-protected continuous cropping system remain limited. In this study, four green manure crops with certain ecological adaptability to plateau environments were selected, namely common vetch, pea, hairy vetch, and radish. Common vetch has relatively strong nitrogen-fixing potential and adaptability to cool environments, which may help improve soil nitrogen supply and enzyme activities. Pea has a relatively well-developed root system and rapid growth and may promote the availability of readily available nutrients. Hairy vetch generally produces high biomass and abundant rhizosphere exudates, which may be more conducive to regulating rhizosphere microbial community composition. Radish has strong root penetration ability, which may help improve soil structure and promote the activation of fixed nutrients [15,19]. These green manure crops differ in morphology, physiology, and ecological functions, providing suitable materials for comparing the mechanisms by which different green manure species regulate soil quality and microbial communities under protected continuous cropping conditions.

Based on the above background, this study proposed the following hypothesis: different green manure species can improve the physicochemical environment of continuously cropped protected soil by altering soil salinity, pH, organic matter, and available nutrient status. Meanwhile, green manure may influence carbon, nitrogen, and phosphorus cycling processes by regulating key soil enzyme activities and microbial community composition, thereby further affecting soil quality. To test this hypothesis, a field experiment was conducted to systematically compare changes in soil physicochemical properties, enzyme activities, and microbial community structure in continuously cropped protected pepper soil under four green manure treatments. We further analyzed the key environmental factors associated with bacterial and fungal community composition and integrated the soil quality index, random forest analysis, and partial least squares path modeling to explore the potential pathways through which green manure affects soil quality. Specifically, this study aimed to address the following questions: first, to determine the effects of different green manure species on soil salinity, nutrient availability, and enzyme activities in continuously cropped protected pepper soil on the Qinghai Plateau; second, to clarify the effects of green manure treatments on soil bacterial and fungal community composition; and third, to identify the key soil factors and microbial ecological indicators closely associated with soil quality improvement. This study may deepen our understanding of green manure–soil–microbe interactions in plateau-protected vegetable fields and provide a theoretical basis and practical reference for selecting suitable green manure species for the improvement of continuously cropped protected soils in Qinghai Province.

## 2. Results

### 2.1. Effects of Different Green Manure Species on Soil Physicochemical Properties and Soil Quality Index

Soil physicochemical properties directly affect crop nutrient uptake and the soil nutrient-retention capacity. As shown in Table 1, the cultivation of different green manure species improved soil physicochemical properties and soil fertility. Compared with CK, all green manure treatments, including L1, L2, L3, and L4, significantly reduced soil total salt content and pH (*p* < 0.05). Among them, L1 resulted in the lowest soil total salt content, reaching only 0.55 g/kg, which represented a decrease of approximately 45.5% compared with CK. Soil pH under the green manure treatments decreased to 8.30–8.42, significantly lower than that of CK at 8.63.

Compared with CK, L1 and L4 markedly increased soil organic matter content by 28.98% and 33.92%, respectively. The highest alkali-hydrolyzable nitrogen content was observed under L1, reaching 80.33 mg/kg, which was 40.93% higher than CK. Compared with CK, available phosphorus content showed increasing trends of 23.72% and 13.27% under L3 and L4, respectively, although the differences were not significant. Soil-available potassium content was higher under all green manure treatments than under CK, although the differences were not consistently significant. Among the treatments, L4 showed the highest mean available potassium content, reaching 265.00 mg/kg.

**Figure 1:**
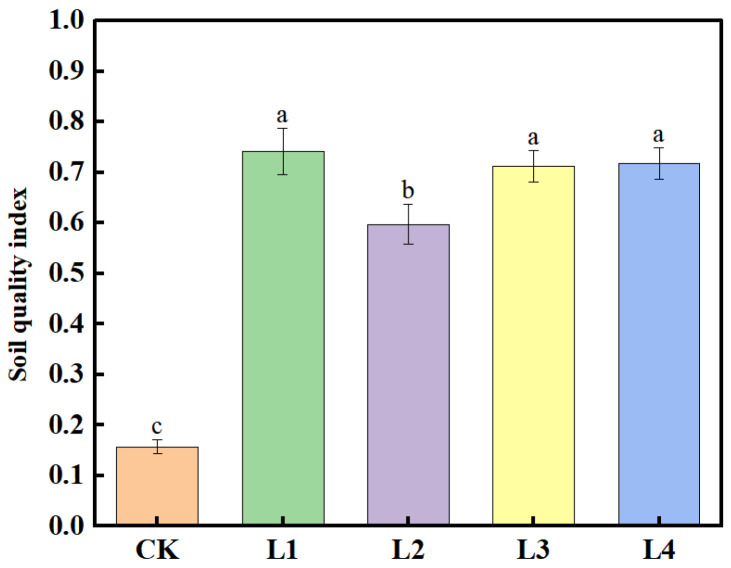
Effects of different green manure treatments on the soil quality index. Different lowercase letters indicate significant differences among treatments (*p* < 0.05).

The soil quality index (SQI) of the 0–20 cm soil layer under different green manure treatments was calculated using six soil physicochemical indicators. A higher SQI value indicates better soil quality. As shown in Figure 1, all green manure treatments significantly increased the SQI compared with CK. The SQI values under L1, L2, L3, and L4 increased to 0.74, 0.60, 0.71, and 0.72, respectively. Among all treatments, L1 showed the highest SQI, with a value of 0.74.

### 2.2. Effects of Different Green Manure Species on Soil Enzyme Activities

**Figure 2:**
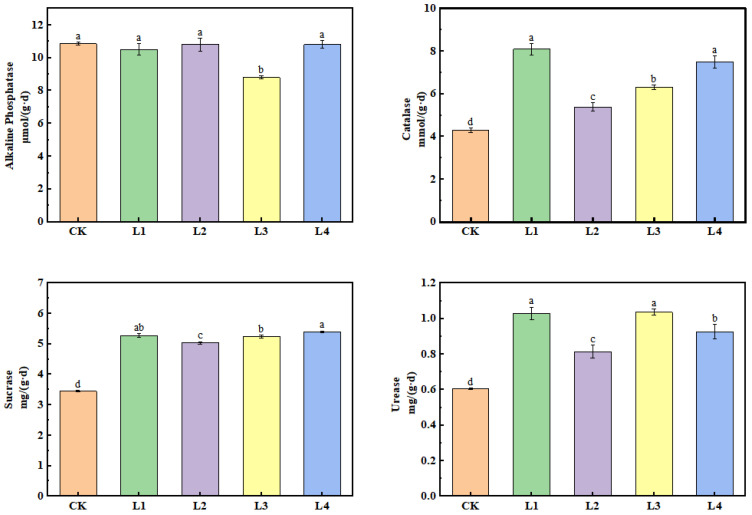
Effects of different green manure treatments on soil enzyme activities. Different lowercase letters indicate significant differences among treatments (*p* < 0.05).

As shown in Figure 2, the activities of soil urease, sucrase, and catalase were significantly higher under all green manure treatments than under the CK. Compared with CK, urease activity increased by 70.49%, 71.32%, and 53.24% under L1, L3, and L4, respectively. Sucrase activity increased by 52.67%, 51.83%, and 56.31%, respectively, while catalase activity increased by 88.47%, 47.02%, and 74.86%, respectively. Differences in alkaline phosphatase activity among treatments were relatively small; however, L3 reduced alkaline phosphatase activity, whereas no significant differences were observed among CK, L1, L2, and L4.

### 2.3. Effects of Different Green Manure Species on Soil Microbial Community Diversity

#### 2.3.1. Effects of Different Green Manure Species on the α-Diversity of Soil Bacterial and Fungal Communities

As presented in Table 2, no significant differences were observed in the Chao1, Shannon, or Simpson indices of soil bacterial and fungal communities between any of the green manure treatments and CK (*p* > 0.05), indicating that short-term green manure cultivation had no marked effect on the overall levels of species richness and evenness.

#### 2.3.2. Effects of Different Green Manure Species on the β-Diversity of Soil Bacterial and Fungal Communities

**Figure 3:**
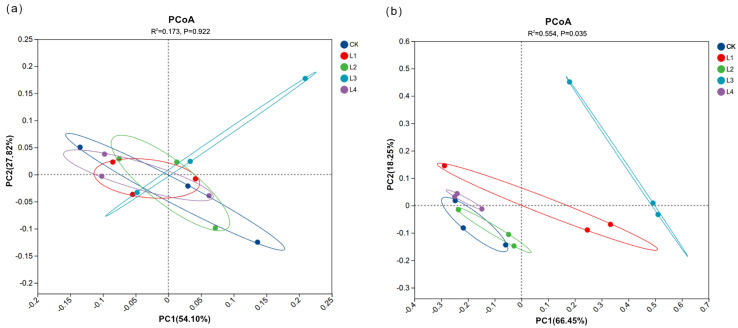
Principal coordinate analysis (PCoA) of soil bacterial (**a**) and fungal (**b**) communities based on Bray–Curtis distances. Effects of Different Green Manure Species on the β-Diversity of Soil Bacterial and Fungal Communities.

Principal coordinate analysis (PCoA) based on Bray–Curtis distance was used to evaluate the effects of different green manure treatments on soil microbial β-diversity. As shown in Figure 3, the PCoA ordination of the bacterial community showed that samples from different treatments were distributed closely and did not exhibit clear separation. The PCoA1 and PCoA2 axes explained 54.10% and 27.82% of the variation in bacterial community composition, respectively, with a cumulative explanation rate of 81.92%. In the ordination plot, shorter distances between sample points indicate greater similarity in community composition. The distribution of different treatments along the coordinate axes suggested that green manure treatments had only a limited effect on soil bacterial β-diversity. This was further supported by the significance test, which showed that green manure treatments had no significant effect on bacterial community composition (R^2^ = 0.173, *p* = 0.922).

For the fungal community, the PCoA ordination showed a more distinct separation among treatments than that observed for the bacterial community. The PCoA1 and PCoA2 axes explained 66.45% and 18.25% of the variation in fungal community composition, respectively, with a cumulative explanation rate of 84.70%. Samples from L3 were mainly clustered on the right side of the PCoA1 axis and were clearly separated from those of the other treatments. In contrast, samples from CK, L2, and L4 were mainly distributed on the left side of the PCoA1 axis and were positioned relatively close to each other, indicating that the fungal community compositions under L2 and L4 were more similar to that under CK and showed no obvious separation. Further significance testing indicated that green manure treatments had a significant effect on fungal community composition (R^2^ = 0.554, *p* = 0.035), suggesting that hairy vetch may be one of the main treatments contributing to changes in soil fungal β-diversity.

#### 2.3.3. Effects of Different Green Manure Species on Soil Bacterial and Fungal Community Composition

**Figure 4:**
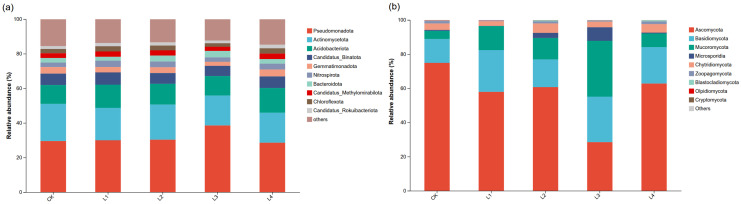
Relative abundances of soil bacterial (**a**) and fungal (**b**) communities at the phylum level.

The composition of soil bacterial communities at the phylum level under different planting patterns is shown in Figure 4a. The dominant bacterial phyla with relatively high abundance were *Pseudomonadota*, *Actinomycetota*, *Acidobacteriota*, *Gemmatimonadota*, *Nitrospirota*, and *Bacteroidota*. Compared with CK, L3 markedly increased the relative abundance of *Pseudomonadota*. The composition of soil fungal communities at the phylum level under different planting patterns is shown in Figure 4b. The dominant fungal phyla with relatively high abundance were *Ascomycota*, *Basidiomycota*, *Mucoromycota*, *Microsporidia*, and *Chytridiomycota*, which together accounted for 97.57–99.12% of the total relative abundance across all treatments. Compared with CK, L1, L2, L3, and L4 reduced the relative abundance of *Ascomycota* and increased the relative abundances of *Basidiomycota* and *Mucoromycota.* In addition, L2 and L3 increased the relative abundance of *Microsporidia,* whereas L1 and L3 reduced the relative abundance of *Chytridiomycota*.

**Figure 5:**
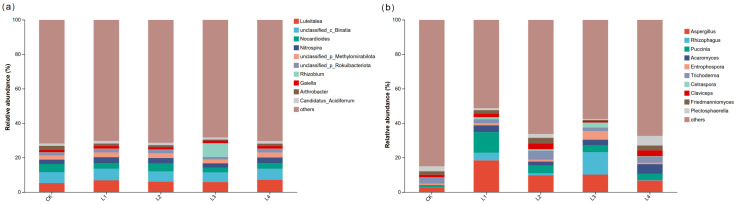
Relative abundances of soil bacterial (**a**) and fungal (**b**) communities at the genus level.

The composition of soil bacterial communities at the genus level under different planting regimes is shown in Figure 5a. The dominant bacterial genera with relatively high abundance were *Luteolalea*, *Nocardioides*, *Nitrospira*, *Rhizobium*, and *Gaiella*. Compared with CK, L3 markedly increased the relative abundance of *Rhizobium*. The composition of soil fungal communities at the genus level under different planting patterns is shown in Figure 5b. The ten dominant fungal genera with relatively high abundance were *Aspergillus*, *Rhizophagus*, *Puccinia*, *Acaromyces*, *Entrophospora*, *Trichoderma*, *Cetraspora*, *Claviceps*, *Friedmanniomyces*, and *Plectosphaerella*, which together accounted for 15.02–48.76% of the total relative abundance across all treatments. Compared with CK, L1, L2, L3, and L4 increased the relative abundances of *Aspergillus*, *Rhizophagus*, *Acaromyces*, *Entrophospora*, and *Cetraspora*. In contrast, L1, L2, and L3 reduced the relative abundance of *Plectosphaerella*, while L3 reduced the relative abundances of *Claviceps* and *Friedmanniomyces*.

### 2.4. Correlation Analysis Between Soil Microorganisms and Soil Physicochemical Properties Under Different Green Manure Species

**Figure 6:**
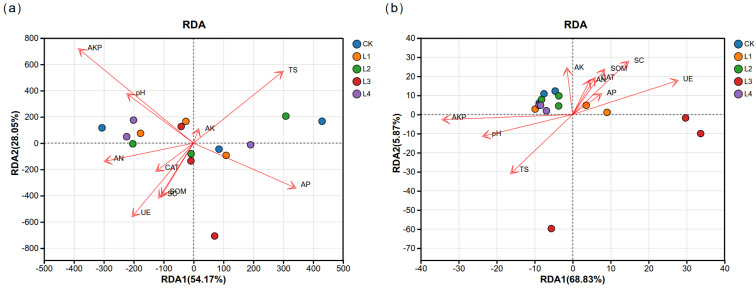
Redundancy analysis (RDA) of soil bacterial (**a**) and fungal (**b**) community structures and environmental factors. TS, total salt; SOM, soil organic matter; AN, alkali-hydrolyzable nitrogen; AP, available phosphorus; AK, available potassium; UE, urease; SC, sucrase; AKP, alkaline phosphatase; CAT, catalase.

The structure of soil microbial communities under different treatments was influenced by multiple environmental factors. As shown in Figure 6a, the cumulative explanatory rate of the RDA for the bacterial community was 82.22%. AKP (R^2^ = 0.4026, *p* = 0.046) significantly influenced bacterial community composition, whereas TS (R^2^ = 0.2559, *p* = 0.187) and CAT showed weaker and non-significant effects. CK, L1, and L4 were mainly located on the left side of the ordination plot and were positively associated with AKP, pH, and AN. In contrast, L2 was mainly distributed on the right side and showed stronger associations with TS and AP. L3 was positively associated with SOM, SC, and UE.

As shown in Figure 6b, the cumulative explanatory rate of the RDA for the fungal community was 74.70%. AKP (R^2^ = 0.4216, *p* = 0.045) and UE (R^2^ = 0.3764, *p* = 0.041) significantly influenced fungal community composition, whereas AP showed a relatively small effect (R^2^ = 0.0480, *p* = 0.05). L3 was clearly separated from CK and the other treatments, showing a strong positive association with UE and negative associations with AKP, pH, and TS.

**Figure 7:**
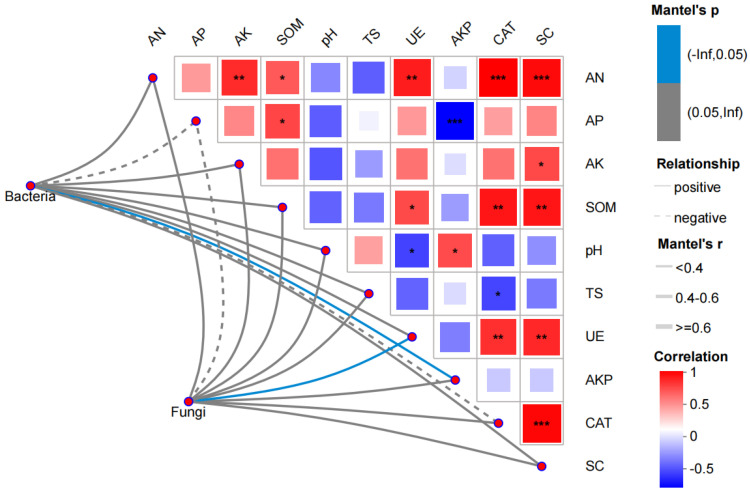
Correlation between soil physicochemical properties and fungal and bacterial communities. Asterisks indicate significance levels (* *p* < 0.05; ** *p* < 0.01; *** *p* < 0.001).

Figure 7 presents the Mantel test results showing the relationships between soil bacterial and fungal community composition and soil physicochemical properties. AN was positively correlated with CAT, SC, and UE, while SOM was strongly positively correlated with CAT and SC activities, suggesting that soil nitrogen and organic matter levels were closely associated with enzyme activities related to carbon and nitrogen cycling. AP was significantly negatively correlated with AKP, suggesting that higher soil-available phosphorus may reduce the microbial demand for alkaline phosphatase production. In addition, soil pH and TS were negatively correlated with most nutrient indicators and enzyme activities, indicating that soil alkalinity and salinity may constrain nutrient availability and enzyme-mediated biochemical processes. The Mantel test further showed that bacterial community composition was mainly associated with AKP, suggesting a potential link between bacterial community variation and soil phosphorus cycling. Fungal community composition was closely associated with UE, indicating that fungal community variation may be related to soil nitrogen cycling.

### 2.5. Regulatory Pathways of Different Green Manure Species on Soil Quality Index

**Figure 8:**
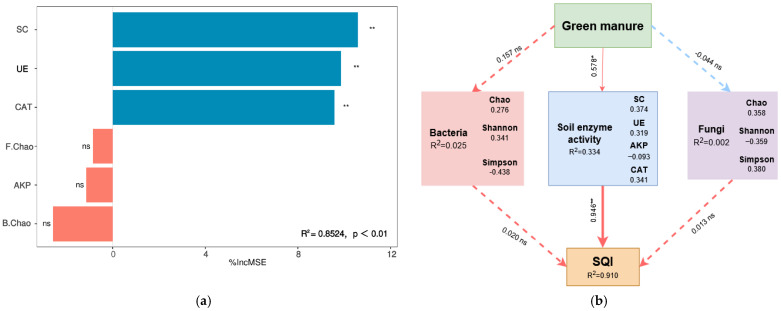
Effects of enzyme activities and microorganisms on SQI: (**a**) random forest model; (**b**) partial least squares path model. Asterisks indicate significance levels (* *p* < 0.05; ** *p* < 0.01) and ns indicates a non-significant effect. In panel (a), blue indicates positive correlations and red indicates negative correlations. In panel (b), blue arrows indicate negative path coefficients, whereas red arrows indicate positive path coefficients. Dashed lines indicate non-significant paths, and solid lines indicate significant paths. The path arrows are labeled with standardized path coefficients and significance levels. R^2^ represents the variance in the dependent variables explained by the model, while the indicators next to the latent variables represent the observed variables.

As shown by the random forest analysis (Figure 8a), SC, UE, and CAT showed relatively high importance values in relation to SQI variation, whereas the Chao1 indices of bacterial and fungal communities and AKP showed relatively low importance. In the partial least squares path model (PLS-PM; Figure 8b), the goodness-of-fit value exceeded 0.600, suggesting an acceptable model fit. The PLS-PM results indicated that green manure cultivation was positively associated with soil enzyme activities, with a path coefficient of 0.578, but showed no significant association with the α-diversity of bacterial or fungal communities. Soil enzyme activities were strongly associated with SQI, with a path coefficient of 0.946, whereas bacterial and fungal α-diversity showed weak and non-significant associations with SQI. The model explained 91.0% of the variance in SQI, and soil enzyme activities appeared to contribute substantially to this explained variation. Within the soil enzyme activity component, the loading coefficients of SC, UE, and CAT were 0.374, 0.319, and 0.341, respectively, whereas AKP showed a minor negative loading. Overall, these results suggest that changes in soil enzyme activities, rather than microbial α-diversity, were more closely associated with SQI variation under different green manure treatments. Given the limited sample size, these findings should be interpreted as exploratory associations rather than direct causal evidence.

## 3. Discussion

### 3.1. Mechanisms by Which Different Green Manure Species Affect Soil Physicochemical Properties and the Soil Quality Index

Long-term continuous cropping-induced soil salinization is one of the major manifestations of soil physicochemical degradation. In this study, all green manure treatments significantly reduced soil total salt content and pH, indicating that green manure cultivation had a certain ameliorative effect on soil salinity and alkalinity under continuous protected cultivation [20]. This effect may be attributed to rhizosphere processes during green manure growth. Specifically, green manure roots may release CO_2_ through root respiration and secrete organic acids and other soluble organic compounds, thereby modifying the rhizosphere acid–base environment. These processes may jointly affect soil salinity and pH by complexing base cations, promoting ion migration, and facilitating plant uptake [21]. In addition, although the soil remained alkaline after green manure treatments, the decrease in pH may have partially alleviated the constraints imposed by alkaline conditions on microbial metabolism and soil enzyme activities, thereby providing a more favorable environmental basis for the subsequent enhancement of soil enzyme activities and shifts in microbial community composition [22].

The soil quality index, calculated based on multiple physicochemical indicators, further indicated that all green manure treatments improved soil quality under continuous protected cultivation, with common vetch showing the best performance. This suggests that, under the present experimental conditions, green manure cultivation can enhance overall soil quality by improving soil salinity, pH, organic matter, and available nutrient status. The effects of different green manure species on soil physicochemical properties varied, which may be related to differences in their biological characteristics, biomass accumulation, nutrient uptake, and nutrient release traits. Common vetch showed pronounced advantages in reducing soil salinity, increasing organic matter, and enhancing alkali-hydrolyzable nitrogen, which may be associated with its leguminous characteristics and relatively strong biological nitrogen-fixing potential [23]. Radish markedly increased soil organic matter and available potassium, possibly due to its well-developed taproot system, which may improve soil structure and facilitate potassium uptake, translocation, and return [24]. Hairy vetch was more conducive to the accumulation of available phosphorus, which may be related to the ability of its root exudates to promote the activation of sparingly soluble phosphorus [25].

### 3.2. Mechanisms by Which Different Green Manure Species Affect Soil Enzyme Activities

Soil enzymes are involved in nutrient decomposition and transformation as well as microbial metabolic processes, and are important indicators of soil quality and the intensity of soil biochemical metabolism [26]. The results of this study showed that all green manure treatments significantly increased the activities of soil urease, sucrase, and catalase, which is consistent with previous findings [27,28]. Sucrase is an important hydrolase involved in soil carbon cycling and can promote the transformation of readily decomposable carbon sources, such as sucrose, into smaller carbon compounds available for microbial utilization [29]. In this study, green manure treatments increased sucrase activity, which may be related to the increase in available organic carbon substrates supplied by green manure root exudates. A greater supply of available carbon sources may stimulate rhizosphere microbial metabolic activity, thereby enhancing enzyme activities associated with carbon transformation [30].

Urease participates in soil nitrogen cycling and is closely related to the soil nitrogen supply capacity [21,31]. In this study, green manure treatments significantly increased urease activity, which may be associated with biological nitrogen fixation by leguminous green manure roots, rhizosphere nitrogen inputs, and enhanced soil nitrogen transformation processes. Catalase is an important enzyme reflecting soil redox status and microbial metabolic activity [32]. The increase in catalase activity under green manure treatments suggests that rhizosphere processes during green manure growth may enhance the potential capacity of the soil system to remove hydrogen peroxide and alleviate oxidative stress, thereby helping to maintain soil microbial activity under saline–alkaline or continuous cropping stress [33].

Phosphorus is an essential nutrient element that limits plant productivity and ecosystem functioning, and its availability is regulated by multiple processes, including mineral weathering, rhizosphere processes, and microbial transformation [34]. In this study, hairy vetch showed the highest soil-available phosphorus content, whereas alkaline phosphatase activity tended to decrease. This phenomenon may be related to feedback regulation of phosphatase synthesis and secretion by soil phosphorus availability. Alkaline phosphatase is mainly involved in organic phosphorus mineralization [35]. Its activity is generally enhanced when soil-available phosphorus is insufficient, thereby promoting the conversion of organic phosphorus into inorganic phosphorus. However, when soil-available phosphorus is relatively high, the demand of microorganisms and plants for phosphatase secretion may decrease, leading to reduced alkaline phosphatase activity. Hairy vetch may increase soil-available phosphorus by secreting low-molecular-weight organic acids through its roots, altering rhizosphere pH, and promoting the activation of sparingly soluble phosphorus, thereby reducing the induction demand for microbial phosphatase production [36,37]. These results suggest that, before incorporation, rhizosphere processes during the growth stage of hairy vetch may contribute to soil phosphorus activation and improved phosphorus availability.

### 3.3. Potential Mechanisms by Which Green Manure Species Regulate Soil Enzyme Activities

The results of this study showed that short-term green manure cultivation significantly altered the β-diversity of the soil fungal community, but had no significant effect on the α-diversity indices of either bacterial or fungal communities. This pattern suggests that soil microbial communities may respond to environmental disturbances in a way whereby community structure is generally more sensitive than species richness indicators [38,39]. It also indicates that short-term green manure treatments may not be sufficient to substantially alter the overall richness of soil microbial communities, but they may already alter microbial community composition by modifying rhizosphere carbon inputs, root exudate composition, and soil physicochemical conditions [30,40].

In this study, the fungal community responded more strongly to green manure treatments than the bacterial community. This may be because fungi grow in the form of mycelia, have relatively slow growth rates and longer generation times, and their community structure may therefore be more readily reshaped when environmental conditions change [41]. In contrast, bacteria are unicellular organisms with rapid division and proliferation rates, which may allow bacterial community composition to remain relatively stable under short-term environmental changes [42]. PCoA analysis showed that the fungal community under hairy vetch differed most clearly from that under CK, which may be related to rhizosphere carbon and nitrogen inputs and root exudate regulation by hairy vetch [26]. In terms of bacterial community composition, hairy vetch significantly increased the relative abundances of *Pseudomonadota* and *Rhizobium*. Root exudates and rhizosphere nutrient conditions associated with green manure may provide suitable ecological niches for some copiotrophic bacteria. *Rhizobium* is commonly associated with symbiotic nitrogen fixation in legumes, and its increased relative abundance may indicate an enhanced potential for soil nitrogen transformation under this treatment [43,44].

From the perspective of fungal community composition, green manure treatments reduced the relative abundance of *Ascomycota* and increased the relative abundances of *Basidiomycota* and *Mucoromycota*. Pathogenic fungi can substantially affect plant growth and yield formation in pepper production; therefore, changes in potentially pathogen-associated taxa deserve attention [44]. Because *Ascomycota* includes several soil-borne pathogenic fungi associated with pepper diseases, the decrease in its relative abundance may indicate a potential reduction in disease-related microbial pressure [45]. However, *Ascomycota* also contains many saprotrophic and endophytic taxa; therefore, this shift should be interpreted cautiously. Some members of *Basidiomycota* and *Mucoromycota* possess strong capacities for organic matter decomposition and nutrient transformation, and their increased relative abundances may be associated with changes in rhizosphere carbon inputs and microbial substrate utilization patterns under green manure cultivation [46].

At the genus level, changes in the relative abundances of *Trichoderma*, *Rhizophagus*, and *Aspergillus* deserve particular attention. *Trichoderma* is generally associated with soil organic matter transformation and antagonistic activity against certain pathogens, and its increased abundance may suggest the potential of green manure treatments to improve the rhizosphere microecological environment [47]. *Rhizophagus*, as a genus of arbuscular mycorrhizal fungi, may theoretically form mutualistic associations with plant roots; its hyphal network can potentially contribute to phosphorus and water acquisition by host plants, thereby supporting plant adaptation under stress conditions [48,49]. *Aspergillus*, as a group containing cellulose-degrading fungi, has a strong capacity for organic substrate utilization and extracellular enzyme secretion, and may participate in rhizosphere organic carbon transformation and nutrient cycling [50]. Meanwhile, the decreased relative abundances of potentially pathogen-associated genera such as *Plectosphaerella* and *Claviceps* may be related to changes in the rhizosphere microenvironment, enhanced resource competition, and the expansion of ecological niches occupied by potentially beneficial taxa.

### 3.4. Regulation of Soil Ecosystem Interactions by Different Green Manure Species

Soil physicochemical properties are important environmental factors affecting microbial growth, metabolic activity, and community composition [51]. Among them, soil organic matter and available nutrients can provide nutritional substrates for microorganisms, thereby further influencing the relative abundance and functional potential of microbial taxa [52]. In this study, redundancy analysis revealed the association patterns between bacterial and fungal communities and soil environmental factors under different green manure treatments, indicating that changes in soil nutrients and enzyme activities induced by green manure cultivation may be closely associated with shifts in microbial community composition [26,53].

At the bacterial community level, CK, common vetch, and radish were positively associated with pH, AKP, and AN, suggesting that these environmental conditions may favor the growth of bacterial taxa adapted to neutral to slightly alkaline conditions [54]. Pea was closely associated with TS and AP, which may indicate the enrichment of bacterial taxa related to salt tolerance and phosphorus utilization under this treatment [55]. Hairy vetch showed strong positive associations with SOM, SC, and UE, suggesting that this treatment may promote the growth of bacterial taxa involved in carbon and nitrogen cycling and organic matter transformation [56]. This finding is consistent with previous studies on continuous cropping systems, which reported that green manure cultivation can restore the activities of extracellular enzymes, such as sucrase and urease, in soils where long-term continuous cropping has reduced carbon and nitrogen transformation efficiency, thereby facilitating nutrient cycling processes [57].

At the fungal community level, CK was mainly distributed in the upper-left region of the ordination plot and was positively associated with pH and TS, suggesting that soil salinity may be an important factor influencing fungal community structure in continuously cropped protected soil [58]. Meanwhile, all green manure treatments showed varying degrees of positive associations with SOM, AN, AP, SC, and CAT, indicating that green manure cultivation may improve fungal community composition by enhancing soil nutrient status and enzyme activities [33]. In particular, common vetch and radish were strongly associated with SOM and SC, which may be related to an increase in available carbon sources and the stimulation of carbon-transformation-related enzyme activities [56]. Pea was positively associated with UE but negatively associated with pH, TS, and AKP, suggesting that this treatment may alleviate soil salinity and alkalinity stress by increasing the organic carbon pool and enhancing nitrogen-cycling enzyme activity, thereby creating a more favorable physicochemical environment for microorganisms [58,59].

Random forest analysis and partial least squares path modeling further showed that SC, UE, and CAT were closely associated with SQI variation, whereas bacterial and fungal diversity and AKP showed relatively limited associations with SQI. This pattern may be related to the specific conditions of the plateau-protected cultivation system, where the recovery of enzyme activities may precede the reconstruction of microbial community diversity under salt accumulation and nutrient limitation [59,60]. Therefore, green manure may regulate soil nutrients and microbial communities by first improving soil salinity, alkalinity, and nutrient status, thereby creating a more suitable physicochemical environment, and then enhancing carbon- and nitrogen-cycling enzyme activities to promote nutrient transformation, which may further influence microbial community composition [61]. Given the limited sample size, the random forest and PLS-PM results should be interpreted only as exploratory evidence of potential association pathways.

From the perspective of sustainable agricultural management, green manure cultivation should not be regarded as a single substitute for chemical inputs, but rather as an ecological regulation component within an integrated management system for continuous cropping obstacles under protected cultivation. In recent years, studies on eco-compatible biocontrol have emphasized that the screening of resistant germplasm, development of bio-derived active compounds, and application of environmentally friendly control measures can reduce reliance on conventional chemical inputs and enhance agroecosystem stability. Therefore, future management of continuous cropping obstacles in protected pepper cultivation on the Qinghai Plateau could further integrate green manure-based soil improvement with disease monitoring, crop resistance evaluation, and eco-friendly pest and disease management practices.

Overall, the improvement effects of different green manure species on continuously cropped soil in this study were mainly reflected in three aspects. First, green manure cultivation improved the soil physicochemical environment by reducing TS and pH and increasing SOM and available nutrient contents. Second, it enhanced biochemical processes related to soil carbon and nitrogen transformation and oxidative stress buffering by increasing SC, UE, and CAT activities. Third, it influenced microbial community composition by modifying the rhizosphere soil microenvironment. These changes may form a potential association pathway of “improved physicochemical properties–enhanced enzyme activities–shifted microbial community composition”, thereby contributing to the improvement of soil quality under continuous protected cultivation.

## 4. Materials and Methods

### 4.1. Experimental Site Description

The experiment was conducted in 2025 in Greenhouse No. 4 of the Shenghang Agricultural Planting Professional Cooperative, Zhiganglaka Village, Cambra Town, Jianzha County, Huangnan Prefecture, Qinghai Province, China (102°15′ E, 36°07′ N). The site is located at an altitude of approximately 2000 m and is characterized by a typical plateau continental semi-arid climate with cool conditions. The mean annual temperature is 7.8 °C, annual sunshine duration reaches 4432 h, the frost-free period is 186 d, and the mean annual precipitation ranges from 350 to 400 mm.

Before the experiment, the basic physicochemical properties of the 0–20 cm cultivated soil layer were as follows: pH, 8.15; soil organic matter, 19.3 g/kg; total salt content, 0.97 g/kg; alkali-hydrolyzable nitrogen, 67.7 mg/kg; available phosphorus, 79.5 mg/kg; and available potassium, 263.3 mg/kg.

### 4.2. Experimental Design

The experiment was arranged in a randomized complete block design with five treatments: CK, common vetch (L1), pea (L2), hairy vetch (L3), and radish (L4). Each treatment was replicated three times, resulting in a total of 15 plots. Each plot measured 2 m × 9 m (18 m^2^), and a buffer strip of 0.5 m × 9 m (4.5 m^2^) was established between adjacent plots to minimize edge effects and reduce potential interference caused by water, nutrient, and root interactions among plots.

Green manure crops were sown on 20 May 2025, and the tested varieties and seeding rates are shown in Table 3. The nutrient contents of the tested green manures are presented in Table 4. No chemical fertilizer was applied during the green manure growth period. Inter-row drip irrigation was used for water management, and irrigation was conducted at the sowing, seedling, branching, and incorporation stages. Greenhouse temperature, humidity, ventilation, and light conditions were managed according to local conventional practices for protected vegetable production. The conventional fertilization regime used for the previous pepper crop was as follows: 3000–4000 kg/mu of decomposed organic manure and 40–50 kg/mu of vegetable-specific compound fertilizer were applied as basal fertilizer; 6–8 kg/mu of drip irrigation fertilizer was applied in stages during the seedling, flowering, full fruiting, and late growth stages.

Soil samples were collected at the full-bloom stage of green manure crops before incorporation. After sample collection, the green manure crops were chopped and incorporated into the soil in mid-July 2025. After approximately 40–50 d of decomposition, pepper seedlings were transplanted in late August.

### 4.3. Sample Collection and Determination

#### 4.3.1. Sample Collection and Processing

Before green manure incorporation, soil samples were collected from the 0–20 cm layer in each plot using a five-point sampling method. The subsamples from each plot were thoroughly mixed to form one composite soil sample. After visible impurities were removed, the samples were air-dried, ground, sieved, and stored in sealed bags for the determination of soil physicochemical properties and enzyme activities.

At the same time, rhizosphere soil samples were collected from randomly selected plants with uniform growth in each plot. During sampling, loosely attached soil around the roots was gently shaken off, and the soil tightly adhering to the root surface was collected as rhizosphere soil. Rhizosphere soil from multiple plants within the same plot was pooled to form one composite rhizosphere soil sample. The samples were placed in sterile 50 mL centrifuge tubes, stored at low temperature in an ice box, transported to the laboratory, and then stored at −80 °C for subsequent microbial sequencing analysis.

#### 4.3.2. Indices Measured and Methods Used

Determination of soil physicochemical properties [62]: Soil pH was measured using a pH meter. Total salt content was determined by the electrical conductivity method. Soil organic matter (SOM) was quantified using the potassium dichromate volumetric method (external heating method). Alkali-hydrolyzable nitrogen (AN) was measured by the alkaline hydrolysis diffusion method. Available phosphorus (AP) was determined by sodium bicarbonate extraction followed by molybdenum–antimony colorimetry. Available potassium (AK) was extracted with ammonium acetate (NH_4_OAc) and quantified using flame photometry.

Determination of soil enzyme activities [63]: Sucrase (SC) activity was assayed using the 3,5-dinitrosalicylic acid colorimetric method. Urease (UE) activity was measured by the sodium phenolate–sodium hypochlorite colorimetric method. Alkaline phosphatase (AKP) activity was determined using the disodium phenyl phosphate colorimetric method. Catalase (CAT) activity was determined by potassium permanganate titration.

Soil microbial genomic DNA was extracted using a genomic DNA extraction kit (D2300; Beijing Solarbio Science & Technology Co., Ltd., Beijing, China). DNA concentration and purity were assessed by 1% agarose gel electrophoresis. The DNA was fragmented using a Covaris M220 ultrasonicator (Covaris, LLC, Woburn, MA, USA), and fragments of approximately 350 bp were selected. Metagenomic sequencing was then performed on the NovaSeq 6000 platform (Illumina, Inc., San Diego, CA, USA).

#### 4.3.3. Calculation Method for the Soil Quality Index

A linear scoring model was used to transform the soil physicochemical indicators into dimensionless scores ranging from 0 to 1. In this study, the “more is better” scoring function was applied for positive indicators (Equation (1)), whereas the “less is better” scoring function was applied for negative indicators (Equation (2)). In the equations, *S_i_* represents the linear score of a given indicator, ranging from 0 to 1; *x_i_* denotes the measured value of the indicator; *x*_max_ represents the maximum value of the indicator; and *x*_min_ represents the minimum value of the indicator [64].

(1) $$S_{i}=\frac{x_{i}-x_{min}}{x_{max}-x_{min}}$$

(2) $$S_{i}=\frac{x_{max}-x_{i}}{x_{max}-x_{min}}$$

To calculate the soil quality index (*SQI*) of the cultivated soil layer (0–20 cm), principal component analysis was first performed on the six soil physicochemical indicators measured in this study. The communality of each indicator was extracted, and the weight of each indicator was calculated as the ratio of its communality to the sum of the communalities of all indicators. The *SQI* was then calculated using Equation (3). A higher *SQI* value indicates better soil quality.

(3) $$SQI=\sum_{i=1}^{n}W_{i}S_{i}$$

In Equation (3), *SQI* represents the soil quality index, *W_i_* represents the weight of the *i*th indicator, *S_i_* represents the score of the *i*th indicator, and *n* represents the number of indicators. In this study, *n* = 6.

### 4.4. Statistical Analysis

Sequencing data were processed and analyzed on the cloud platform provided by Shanghai Majorbio Bio-pharm Technology Co., Ltd. (Shanghai, China; https://cloud.majorbio.com/, accessed on 6 November 2025). Raw metagenomic sequencing data were first subjected to quality control to remove adapter sequences, low-quality reads, and reads containing a high proportion of ambiguous bases, thereby generating high-quality clean reads. The clean reads were then used for metagenomic assembly, followed by open reading frame (ORF) prediction based on the assembled sequences. The predicted gene sequences were dereplicated to construct a non-redundant gene catalogue. DIAMOND software (University of Tübingen, Tübingen, Germany; http://ab.inf.uni-tuebingen.de/software/diamond/, accessed on 6 November 2025) was used to align the non-redundant gene catalogue against the non-redundant protein database (NR), and microbial taxonomic annotation information was obtained. Based on the taxonomic annotation results, bacterial and fungal abundance matrices were separately extracted from the same shotgun metagenomic dataset for subsequent analyses of community composition and diversity.

To minimize the influence of differences in sequencing depth among samples, the bacterial and fungal abundance matrices were standardized separately before diversity analyses. Community composition analysis was performed based on relative abundance matrices, in which the relative abundance of each taxon was calculated using the total reads annotated to the corresponding microbial group in each sample as the denominator. Chao1, Shannon, and Simpson indices were calculated using the algorithms implemented in mothur software (version 1.30.2; https://mothur.org/wiki/calculators/, accessed on 6 November 2025) and R software (version 3.3.1; R Core Team, Vienna, Austria) to evaluate the richness, diversity, and evenness of soil bacterial and fungal communities. Principal coordinate analysis (PCoA) based on Bray–Curtis dissimilarity matrices was performed to compare differences in microbial community structure among treatments. The effects of different green manure treatments on bacterial and fungal community composition were tested using permutational multivariate analysis of variance (PERMANOVA) with the adonis2 function in the vegan package in R software (version 4.0.2; R Core Team, Vienna, Austria). The number of permutations was set to 999, and R^2^ was used to indicate the proportion of variation in community composition explained by the treatment.

Microbial community composition was analyzed at the phylum and genus levels, and taxa were ranked according to their mean relative abundance across treatments. The Kruskal–Wallis H test was used to identify differentially abundant taxa among treatments. Redundancy analysis (RDA) was performed to analyze the relationships between soil bacterial and fungal community composition and soil physicochemical properties and enzyme activities. Mantel tests were used to examine the correlations between microbial community dissimilarity and environmental factors. The results of RDA and Mantel tests were used mainly to interpret associations among variables and were not considered evidence of direct causal relationships.

Data were organized using Excel 2019, and figures were generated using Origin 2021 and the ggplot2 package in R software (version 4.0.2; R Core Team, Vienna, Austria). Soil physicochemical properties, enzyme activities, and the soil quality index (SQI) were statistically analyzed using SPSS 27.0. Before analysis of variance, all data were tested for normality using the Shapiro–Wilk test and for homogeneity of variance using Levene’s test. Data satisfying the assumptions of normality and homogeneity of variance were analyzed using one-way analysis of variance (one-way ANOVA), followed by Duncan’s multiple range test to compare differences among green manure treatments. Statistical significance was set at *p* < 0.05.

Random forest models were constructed using the randomForest, rfUtilities, and rfPermute packages in R to identify important explanatory variables closely associated with SQI variation. During model construction, the random seed was set to 123 to ensure reproducibility; the number of decision trees (ntree) was set to 500, and the variable selection parameter (mtry) was set to the default value. Model significance was evaluated using 99 permutation tests, and variable importance was calculated based on 100 repeated permutations. The increase in mean squared error (%IncMSE) was used to evaluate the importance of each explanatory variable.

Partial least squares path modeling (PLS-PM) was used to analyze potential association pathways among green manure treatment, soil enzyme activities, microbial α-diversity, and SQI. PLS-PM was performed using the plspm package in R. In the model, green manure treatment was set as the exogenous variable, whereas soil enzyme activities, bacterial α-diversity, fungal α-diversity, and SQI were set as latent variables. Soil enzyme activity was represented by sucrase, urease, alkaline phosphatase, and catalase activities. Bacterial and fungal α-diversity were each represented by the Chao1, Shannon, and Simpson indices. Model performance was evaluated using goodness of fit (GOF), path coefficients, and the coefficients of determination (R^2^) of endogenous variables. Given the limited sample size of this study, the random forest and PLS-PM results were used mainly for variable importance ranking and exploratory analysis of potential association pathways, rather than as evidence of strict causal relationships.

## 5. Conclusions

This study showed that short-term green manure cultivation exerted certain ameliorative effects on continuously cropped protected pepper soil on the Qinghai Plateau during the growth stage before incorporation. All four green manure treatments significantly reduced soil total salt content and pH, increased soil organic matter, alkali-hydrolyzable nitrogen, available potassium, and other nutrient levels, and improved the SQI to varying degrees. Meanwhile, green manure treatments significantly increased soil urease, sucrase, and catalase activities, indicating that rhizosphere processes during green manure growth may enhance biochemical processes related to soil carbon and nitrogen transformation and alleviate saline–alkaline stress to some extent. Among the green manure species, common vetch performed better in reducing soil salinity, increasing alkali-hydrolyzable nitrogen, and improving SQI, whereas hairy vetch was more conducive to available phosphorus accumulation and changes in fungal community structure.

Microbial community analysis showed that short-term green manure cultivation did not significantly alter bacterial or fungal α-diversity, but significantly affected fungal β-diversity and community composition, suggesting that fungal community structure was more sensitive to changes in the rhizosphere environment induced by green manure. Alkaline phosphatase activity showed a decreasing trend under some treatments, which may be related to the reduced demand for phosphatase secretion by microorganisms and plants after the increase in soil-available phosphorus. Integrated results from RDA, Mantel tests, random forest analysis, and partial least squares path modeling indicated that soil salinity, pH, available nutrients, and enzyme activities were closely associated with changes in microbial community composition and SQI. Among these factors, sucrase, urease, and catalase were important variables closely related to SQI variation. Overall, different green manure species may affect soil quality under continuous protected cultivation through a potential association pathway of “improved physicochemical properties–enhanced enzyme activities–shifted microbial community composition”.

It should be noted that this study still has several limitations. Root exudate composition, microbial functional gene expression, crop yield, and disease incidence were not directly measured, and the long-term effects after green manure incorporation and continuous multi-year application were not evaluated. Therefore, this study does not suggest that a single season of green manure cultivation alone can fully resolve continuous cropping obstacles in protected pepper production. Rather, it indicates that green manure can serve as an important measure for soil improvement and microecological regulation in plateau-protected cultivation systems. Future studies should integrate long-term field trials, crop yield and quality assessments, disease investigations, and related analyses to further clarify the long-term effects of green manure on alleviating continuous cropping obstacles in protected pepper cultivation on the Qinghai Plateau and its underlying microbial functional mechanisms, thereby providing a more reliable theoretical basis and management strategy for the sustainable production of plateau-protected vegetables.

## Figures and Tables

**Table 1:** Effects of different green manure treatments on soil physical and chemical properties.

Treatment	Total Salt /(g/kg)	pH	Organic Matter /(g/kg)	Alkali-Hydrolyzable Nitrogen/(mg/kg)	Available Phosphorus /(mg/kg)	Available Potassium /(mg/kg)
CK	1.01 ± 0.12 a	8.63 ± 0.07 a	12.97 ± 0.40 b	57.00 ± 2.52 c	64.80 ± 2.63 a	212.00 ± 23.26 b
L1	0.55 ± 0.05 b	8.34 ± 0.11 b	16.73 ± 1.45 a	80.33 ± 6.51 a	69.03 ± 11.94 a	256.67 ± 7.09 a
L2	0.56 ± 0.08 b	8.38 ± 0.06 b	15.03 ± 0.97 ab	68.00 ± 4.00 b	67.20 ± 12.77 a	256.33 ± 10.07 a
L3	0.59 ± 0.06 b	8.30 ± 0.08 b	16.20 ± 2.02 a	75.00 ± 8.72 ab	80.17 ± 7.11 a	249.33 ± 11.59 a
L4	0.62 ± 0.07 b	8.42 ± 0.10 b	17.37 ± 0.86 a	76.67 ± 3.21 ab	73.40 ± 2.17 a	265.00 ± 22.54 a

Note: CK: Control group; L1: common vetch; L2: pea; L3: hairy vetch; L4: radish. Different lowercase letters indicate significant differences among treatments (*p* < 0.05). The same as below.

**Table 2:** Soil microbial alpha diversity indices.

Microbial Types	Treatment	Chao1	Shannon	Simpson
Bacterial	CK	2163 ± 32.42 a	6.27 ± 0.29 a	0.014 ± 0.005 a
	L1	2182 ± 24.00 a	6.29 ± 0.21 a	0.014 ± 0.003 a
	L2	2216 ± 44.91 a	6.38 ± 0.22 a	0.012 ± 0.003 a
	L3	2180 ± 51.85 a	6.25 ± 0.24 a	0.013 ± 0.002 a
	L4	2165 ± 62.34 a	6.20 ± 0.21 a	0.014 ± 0.003 a
Fungal	CK	183.67 ± 28.54 a	4.51 ± 0.08 a	0.02 ± 0.003 a
	L1	200.00 ± 30.32 a	4.29 ± 0.41 a	0.03 ± 0.018 a
	L2	188.33 ± 27.30 a	4.55 ± 0.13 a	0.02 ± 0.003 a
	L3	284.33 ± 72.39 a	4.31 ± 0.41 a	0.03 ± 0.019 a
	L4	179.00 ± 15.13 a	4.58 ± 0.24 a	0.02 ± 0.009 a

Note: Different lowercase letters indicate significant differences among treatments within the same microbial group and index (*p* < 0.05).

**Table 3:** Types and seeding rates of green manure.

Treatment	Variety	Seeding Rate (kg/m^2^)	Returned Amount (kg/m^2^)
CK		0	0
L1	Ximu 333	0.012	2.0
L2	Longwan No. 2	0.150	0.9
L3	Qingtiao No. 1	0.006	2.5
L4	Aizaoluo No. 1	0.004	7.3

**Table 4:** Nutrient contents of tested green manures.

Treatment	Total Nitrogen (g/kg)	Total Phosphorus (g/kg)	Total Potassium (g/kg)
CK	0	0	0
L1	32.60	5.09	50.64
L2	40.50	5.44	52.43
L3	37.16	5.35	54.00
L4	35.72	8.44	67.45

## Data Availability

The original contributions presented in this study are included in the article. Further inquiries can be directed to the corresponding author.
